# Comparative characterisation of extracellular vesicles from canine and human plasma: a necessary step in biomarker discovery

**DOI:** 10.1007/s11259-024-10405-0

**Published:** 2024-05-08

**Authors:** Stephanie Marie Bollard, J. Howard, C. Casalou, L. Mooney, S. Peters, C. Sweeney, A. Ajaykumar, K. Triana, A. McCann, P. A. Kelly, S. M. Potter

**Affiliations:** 1 UCD School of Medicine, University College Dublin, Belfield, Dublin 4, Ireland; 2 UCD Conway Institute of Biomolecular and Biomedical Research, University College Dublin, Belfield, Dublin 4, Ireland; 3 Department of Plastic & Reconstructive Surgery, Mater Misercordiae University Hospital, Eccles Street, Dublin 7, Ireland; 4 UCD Charles Institute of Dermatology, University College Dublin, Belfield, Dublin 4, Ireland; 5 UCD School of Veterinary Medicine, University College Dublin, Belfield, Dublin 4, Ireland; 6 UCD Clinical Research Centre, University College Dublin, Belfield, Dublin 4, Ireland

**Keywords:** Extracellular vesicles, Size exclusion chromatography, Comparative medicine, OneHealth, Canine

## Abstract

Extracellular Vesicles (EV) have become an interesting focus as novel biomarkers of disease and are increasingly reported upon in humans and other species. The Minimal Information for Studies of Extracellular Vesicles 2018 (MISEV2018) guidelines were published to improve rigor and standardisation within the EV field and provide a framework for the reliable isolation and characterisation of EV populations. However, this rigor and standardisation has been challenging in the area of comparative medicine. Herein we present the successful isolation of EVs from human and canine plasma using Size Exclusion Chromatography and characterise these EVs according to best international practice. This study provides evidence for the reliable comparison of human and canine EVs isolated by this approach, and a baseline description of the EVs from healthy dogs to inform future biomarker studies. This work also demonstrates that the MISEV2018 guidelines can be successfully applied to EVs isolated from canine plasma.

**Supplementary Information:**

The online version contains supplementary material available at 10.1007/s11259-024-10405-0.

## Introduction

Circulating small Extracellular Vesicles (sEVs), measuring 30–150 nm, are potential biomarkers of disease, as their composition and cargo reflect their cell of origin (Yuana et al. 2013). Standardisation efforts in the EV community through the development of the Minimal Information for Studies of Extracellular Vesicle 2018 (MISEV2018) guidelines (Thery et al. 2018) have resulted in improved reproducibility and reliability of published EV studies, and methods for isolation and characterisation of human EVs are well described.

As we seek to reduce, refine, and replace laboratory-based animal models of disease, the ability to study spontaneous diseases across species in Comparative Medicine is being utilised as a complementary alternative to animal studies. Extracellular Vesicle studies involving non-human species are increasing, with studies reporting EV isolation from the plasma of species such as dogs, horses, pigs, cats, amongst others (Eirin et al. 2020; Almiñana et al. 2021; Howard et al. 2022; Kulka et al. 2022; Moccia et al. 2023). Dogs have been presented as an ideal comparative medicine model, due to their similar environment, nutrition, aging and genetic factors (Gordon et al. 2009). As a result, many authors have recently published on canine EVs, and alluded to their potential human comparability (Zmigrodzka et al. 2019; Kulka et al. 2022; Moccia et al. 2023). However, few canine EV studies have successfully fulfilled the characterisation guidelines as outlined in MISEV2018, despite the recommendation that *‘the need to demonstrate presence of EV markers and absence or depletion of putative contaminants… can be generalised to all species, cells and conditions’ *(Thery et al.  2018), and the wide support of the MISEV2018 guidelines amongst the EV community (Witwer et al. 2021).

In this report, we present the successful isolation and characterisation of plasma derived EVs from canine and human plasma using SEC, according to the MISEV2018 guidelines. This work provides a framework for other canine EV based comparative studies.

## Materials and methods

### Recruitment and plasma preparation

Canine plasma samples were obtained from 10 healthy dogs undergoing routine veterinary care, once all clinical diagnostics were complete, in accordance with the protocol approved by the Animal Research Ethics Committee, University College Dublin (UCD; Ref: AREC-E-20-26-Kelly). No samples from dogs were taken specifically for this study, and all dogs remained in the care of their owners throughout. The samples were pooled to enable the replication of experiments across technical replicates, given the constraint of very small volumes remaining after clinical diagnostics. The pooled plasma was then aliquoted and stored at -80 °C.

Healthy human controls (Supplementary Table 1) were recruited from patients attending the outpatient Department at the Mater Misericordiae University Hospital, Dublin, Ireland, following Institutional Review Board ethical approval (Ref: 1/378/2189). Full informed consent was obtained, and a EDTA plasma sample was obtained and spun at 3000x*g* for 10 min to separate plasma, and subsequently at 2500x*g* to remove platelets. Platelet poor plasma was then aliquoted and stored at -80 °C for subsequent Extracellular Vesicle (EV) isolation.

### Extracellular vesicle (EV) isolation

EVs were isolated from plasma, using Size Exclusion Chromatography (SEC), using iZon qEVoriginal Gen2 70 nm Columns (iZon Science). Isolations from both human and canine plasma were performed using an Automated Fraction Collector (iZon Science). For each isolation, the column was flushed with freshly filtered PBS, and 500µL plasma was added to the loading frit. A buffer volume of 100µL was allowed to pass before collection of 13 fractions each of 400µL elute were collected for downstream analysis.

### Nanoparticle tracking analysis (NTA)

Nanoparticle Tracking Analysis (NTA) was performed using a NanoSight NS300, to determine EV size and concentration from the individual EV fractions. The NTA instrumentation was configured with a 488 nm laser and a high sensitivity CMOS camera. Samples were diluted in freshly filtered PBS (1:25 dilution) and analysed under constant flow conditions (flow rate = 50) at 25 °C, camera level 10–12 and screen gain 5. Five x 60 s videos were captured for each sample, and human and canine EVs were analysed in triplicate. Data were analysed using NTA 4.1 software, with a detection threshold of 10 and bin size of 2.

### EV lysis and protein quantification

EVs were lysed prior to establishing the protein concentration in each SEC fraction. A volume of 50µL elute from each fraction were collected and lysed with 10µL of a Triton-based lysis buffer [50mM Tris HCL pH 7.4, 150mM NaCl, 1% Triton, 1% EDTA, 1mM phenylmethylsulphonyl, 1% phosphatase inhibitor, 1% protease inhibitor cocktail]. Samples were incubated on ice for 30 min, vortexing every 10 min, followed by water bath sonication for 3 min and centrifugation at 10,000x*g* for 20 min. Protein quantification was then performed using the Pierce BCA Protein Assay (ThermoFisher, #23,227) kit in duplicate according to manufacturer’s instructions, with concentration determined from a Bovine Serum Albumin (BSA) standard curve.

### Transmission electron microscopy

Once the optimal purified collection volume (PCV) of the plasma EVs had been determined, four EV fractions were pooled for further analysis of morphology using TEM. From the PCV, 400µL of EVs were then concentrated using a 10 kDa cut-off centrifugal filter (Amicon, #UFC501096). 10µL of EVs were placed on a formvar carbon-coated copper EM grid for 60 min. Vesicle coated grids were washed three times with PBS and then fixed using 2.5% glutaraldehyde for 10 min. The grids were then washed in distilled water, before staining with 2% uranyl acetate for 15 min, and embedded in methyl cellulose-UA for 10 min on ice. Excess cellulose was removed, and the grids allowed to air dry. TEM was then performed using a FEI Tecnai 120 microscope, operating at an accelerating voltage of 120 kV. Images were taken of the entire field at 87000X.

### Western blot analysis for extracellular vesicle markers

EV isolations were performed as described in triplicate from the pool of canine plasma and from three individual human controls, collecting the optimal PCV. These were lysed and protein quantified as described above. A concentrate of 30 µg of protein was combined with 5µL 1xSDS loading buffer (New England BioLabs) and 1.25 M DTT and heated at 95 °C for 5 min. Proteins were run on an 8–12% SDS NuPAGE Bis-Tris gel (Invitrogen, #NP0321BOX) in a NuPAGE MOPS SDS Running buffer (Invitrogen, #NP0001) at 200 V for 42 min. Resolved proteins were then transferred to a nitrocellulose membrane at a constant 50 V for 80 min. The effective transfer was confirmed using a Ponceau stain, and membranes were then blocked in 1x TBS containing 5% (w/v) BSA. Proteins were detected by incubation with primary antibodies; Alix (abcam, ab186728, 1/1000), Calnexin (abcam, ab 112,995, 1/2000), HSP70 (abcam, ab181606, 1/1000), CD63 (abcam, ab271286, 1/1000), ApoA1 (abcam, ab211472, 1/100) in blocking solution overnight at 4 °C. Membranes were then washed in TBS with 0.1% Tween, and incubated in the appropriate species IRDye-conjugated secondary antibody (Li-COR Biosciences, IRDye-680RD conjugated goat anti-rabbit IgG #925-68071 and IRDye-800CW goat anti-mouse IgG #925-32210) for 1 h in the dark at room temperature. Proteins were then visualised by scanning the membrane with an Odyssey CLX Infrared Imaging System (Li-COR Biosciences) and processed using Image Studio Lite (Li-COR Biosciences, v5.2.5).

### Data representation

Data were collated and analysed using GraphPad Prism (v9.4.1).

## Results

### Determination of the optimal purified collection volume to allow comparison of canine and human EVs

**Fig. 1:**
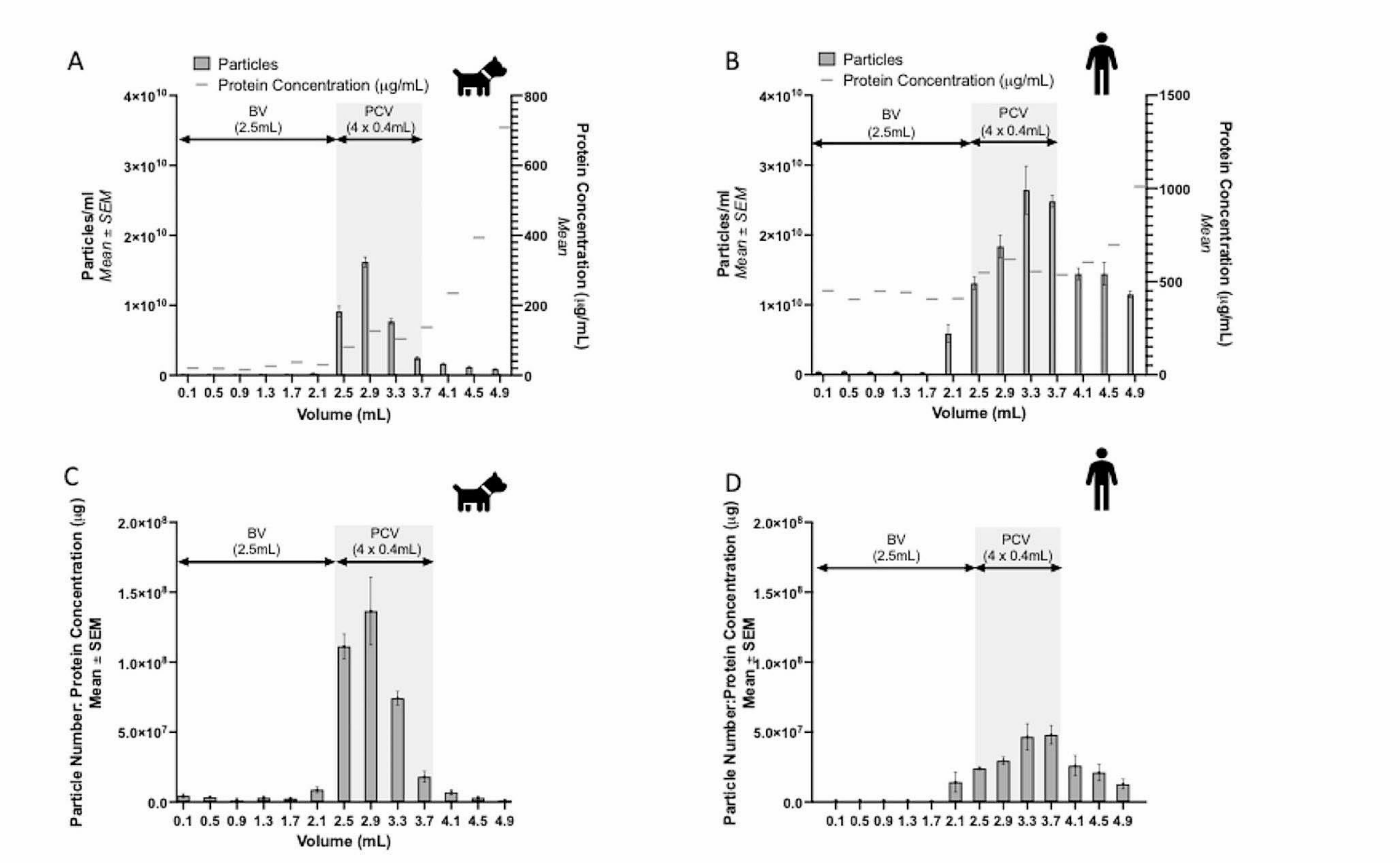
Comparison of the elution profile of canine and human EVs isolated using SEC. Nanoparticle Tracking Analysis (NTA) and BCA protein assay were used to compare the elution profile of EVs isolated from 500µL plasma from the pooled plasma of *n*  = 10 dogs and a single healthy human, each across *n*  = 3 technical replicates. NTA demonstrates the concentration of EVs in each fraction isolated using the qEV Gen2 70 nm Izon column in (**A**) canine and (**B**) human plasma, with the majority of EVs eluting in the Purified Collection Volume (PCV) consisting of 4 fractions, following a Buffer Volume (BV) of 2500µL. Subsequent fractions showed an increase in protein concentration, indicating contamination. Purity examination using particle per µg protein concentration ratio, confirm the selected fractions are the least contaminated in (**C**) canine and (**D**) human samples

Nanoparticle Tracking Analysis (NTA) and protein quantification using the Bicinchoninic Acid (BCA) protein assay allowed optimisation of the collection schedule from the iZon Gen2 qEVoriginal Size Exclusion Chromatography (SeC) column to enrich for small Extracellular Vesicles (sEVs) from canine and human plasma (Fig. 1). Peak concentration of particles was seen in the elute collected in the Purified Collection Volume (PCV) consisting of the 4 × 400µL fractions collected following a buffer volume of 2500µL. Protein concentration in the subsequent fractions isolated from both species increased, indicating co-isolation of protein contaminants. This is further supported upon examination of the purity of individual fractions as seen in Fig. 1c and d, using a ratio of the number of particles as measured on NTA per µg of protein, in which a higher ratio is suggestive of a higher degree of purity (Webber and Clayton 2013).

### Comparison of canine and human EV morphology by TEM

**Fig. 2:**
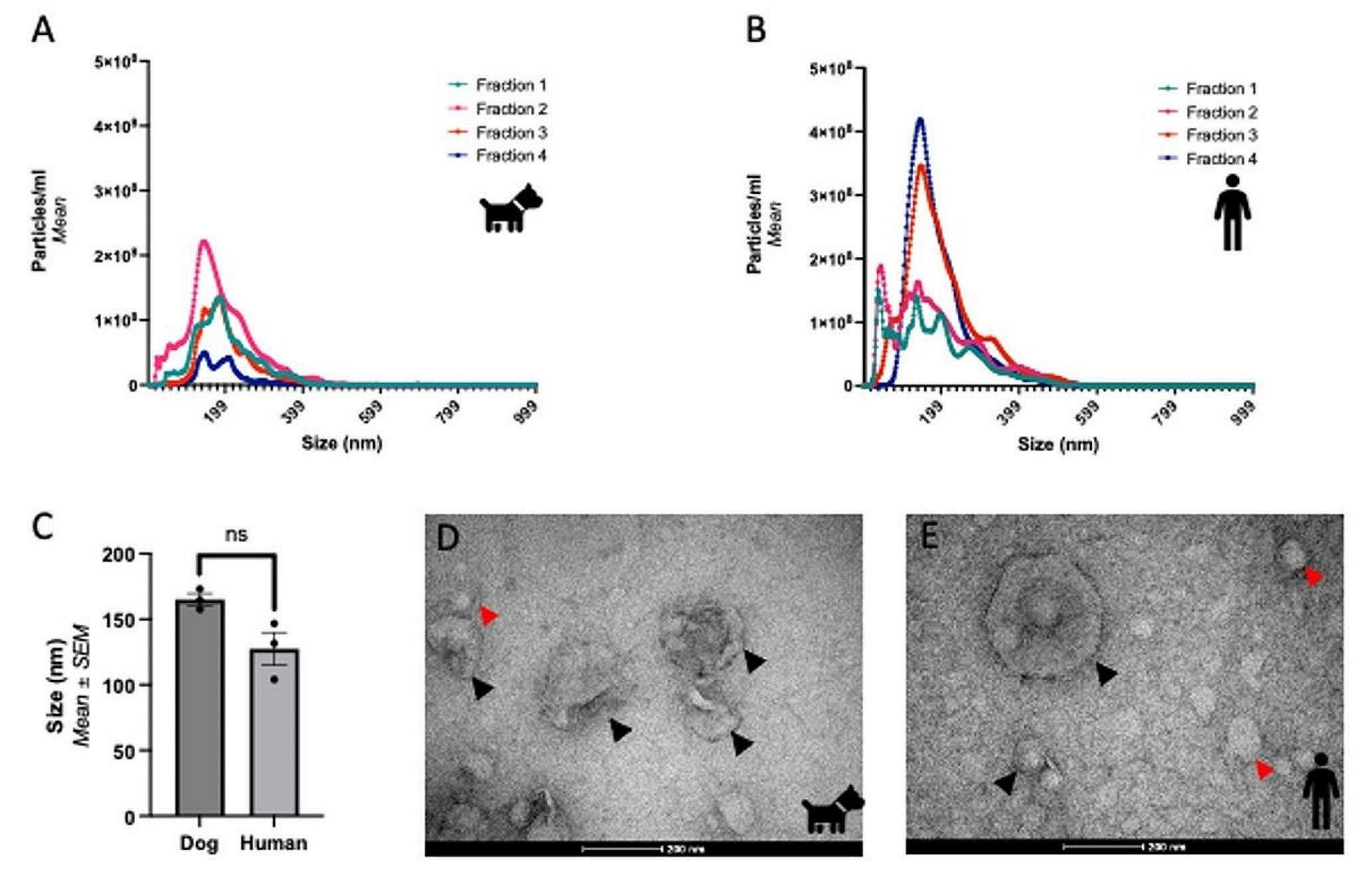
Size Exclusion Chromatography successfully isolates and enriches for small EVs (<200nm) from canine and human plasma.  The size distribution curves of the numbered fractions from the purified collection volume as measured using NTA are shown from (**A**) canine and (**B**) human plasma. Both species demonstrate a similar size distribution profile across the selected fractions, though a higher volume of small EVs (< 200 nm) was eluted in Fraction 2, after a buffer volume of 2500µL in the canine EVs (**A**) whereas the majority of small EVs (200 nm) were eluted in Fraction 3 and 4 for the human sample (**B**). Similarly, (**C**) the modal size of the EVs isolated within the purified collection volume was similar ( *p* = 0.10, Mann-Whitney test ) and < 200 nm in both species. TEM images taken at 87000x demonstrate the expected cup shape morphology of the EVs isolated from both (**D**) dogs and (**E**) human. In these images, the EVs as indicated by the black arrows are of the expected size, approximately 200 nm. Lipoproteins are indicated by red arrows and can be seen in the preparations from both species. Wide-field images of the EVs of both species are seen in Supplemental Fig. 2

Size distribution profiles of the PCV fractions of both species demonstrated that the elute is predominantly composed of particles < 200 nm in size, with particles > 200 nm contributing a smaller concentration, indicating successful enrichment for sEVs (Fig. 2). The PCVs isolated from dogs and humans consisted of particles with a mean modal size of 127.5 ± 21.6 nm and 165.0 ± 7.8 nm respectively (*p* = 0.10, Mann-Whitney test). Transmission Electron Microscopy demonstrates the presence of particles with a typical cup-shaped morphology and size consistent with EVs from both species. Small (< 200 nm) and larger (> 200 nm) EVs can be seen (black arrows), as well as lipoproteins (red arrows).

### Characterisation of canine and human EVs using recognised EV markers

**Fig. 3:**
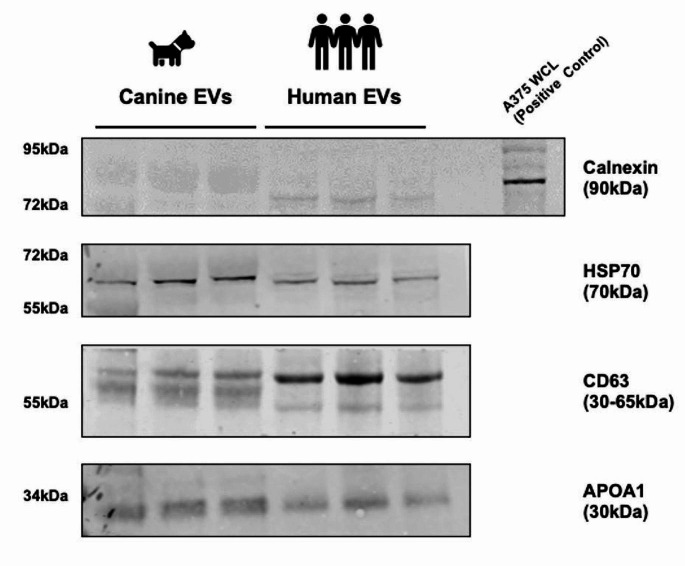
Comparative Western Blot analysis of isolated canine and human EVs showing similar EV marker profile. EVs isolated from pooled plasma of dogs (*n*  = 10) in triplicate and individual human (*n*  = 3) donors were lysed, concentrated, and analysed using western blot according to the MISV2018 guidelines. All EVs were positive for HSP70, CD63 and ApoA1, and negative for calnexin, as expected for small EVs < 200 nm isolated using SEC. Representative images are shown

Extracellular Vesicles were isolated from the pooled plasma collected from *n* = 10 healthy dogs and from individual samples from three healthy humans. These EVs were then concentrated using centrifugal filtration, and presence and absence of relevant EV markers were analysed by Western Blot (Fig. 3). The presence of CD63, a tetraspanin and non-tissue specific transmembrane protein, and HSP70, a cytosolic protein promiscuously incorporated in EVs, are both clearly demonstrated in the EV preparations from both species. The absence of calnexin, a protein associated with the Endoplasmic Reticulum and Golgi apparatus and not enriched in the small EV populations originating from Multivesicular Bodies, supports the assertion that the PCV is enriched for small EVs < 200 nm. However, the presence of non-EV co-isolated structures and contaminants is also apparent in both species, as demonstrated by the presence of ApoA1.

## Discussion

Our results show the successful isolation and characterisation of Extracellular Vesicles (EVs) from canine and human plasma, in accordance with the Minimal Information for Studies of Extracellular Vesicle 2018 (MISEV2018) guidelines. Similarly, this also demonstrates that EVs isolated from both species using Size Exclusion Chromatography (SEC) can be reliably compared, as they demonstrate similar elution and size profiles, and can be profiled using accepted characteristic EV markers.

Dogs are a popular species for comparative medicine and oncology studies (Paoloni and Khanna 2007). As EV formation and release appears to be evolutionarily conserved between species (Lawson et al. 2017), EVs have become an attractive focus for novel biomarkers of disease in comparative studies. However, the lack of availability of species-specific antibodies and the small volume of available biological samples obtainable from some species (Lawson et al. 2017; Howard et al. 2022) have made the direct comparison of canine and human EVs challenging to date. However, when attempting to address these challenges, it is important to also consider species-specific factors. For instance, the differential bands observed in the Western blot of CD63 between humans and dogs (Fig. 3) suggest the influence of additional variables, such as differential glycosylation (Tominaga et al. 2014).

Lipoproteins were co-isolated with the EVs from both species when using SEC, as seen in the TEM (Fig. 2) and Western Blot (Fig. 3) images presented. Successful removal of lipoprotein contaminants from plasma EVs remains a challenge for researchers in the field (Thery et al. 2018, Takov et al. 2019). Despite this, SEC is commonly used in plasma-derived EV isolation (Royo et al. 2020) as it is reported to minimally alter the EV preparation preserving the functionality of EVs (Mol et al. 2017), and co-isolate less other soluble factors (Wallis et al. 2021). In addition, it is inexpensive, quick and requires minimal additional equipment, making it a strong candidate for biomarker discovery and eventual clinical translation (Veerman et al. 2021).

Nonetheless, the presence of these contaminants highlights the necessity of EV characterisation when preforming EV population studies. Whilst many groups have successfully isolated canine EVs using various isolation methods, few have fully characterised them, with many studies reporting features such as positive markers and concentration, without an assessment of preparation purity (Kuwahara et al. 2021; da Silva Nunes et al. 2022; Luu et al. 2022). It has been demonstrated that this assessment of non-EV co-isolated components is the least reported upon in the EV literature in general (Poupardin et al. 2021), with the veterinary and comparative biology disciplines being no exception. However, non-EV co-isolated structures can be falsely detected as EVs in many common quantification methods such as NTA (Brennan et al. 2020) and may alter results of functional studies (Takov et al. 2019). As a result, it is essential to include an assessment of these contaminants to inform the purity and reliability of a preparation before any conclusions about the actions or influence of the EVs may be drawn.

## Conclusions

In conclusion, we have fully characterised the Extracellular Vesicles (EVs) isolated from the plasma of healthy dogs and human donors, according to the Minimal Information for Studies of Extracellular Vesicle 2018 (MISEV2018) guidelines. For the first time, we have demonstrated that, following EV isolation with the clinically applicable method of Size Exclusion Chromatography (SEC), canine and human EVs can be compared. This will allow future comparative studies to replicate these methods and accurately describe EV populations, as well as providing a useful baseline for the future study of EVs isolated from the plasma of dogs with different pathologies.

### Electronic Supplementary Material

Below is the link to the electronic supplementary material.

Supplementary Material 1

## Data Availability

No datasets were generated or analysed during the current study.
