# Supplementary material for: Comparative characterisation of extracellular vesicles from canine and human plasma: a necessary step in biomarker discovery
Source: Vet Res Commun. 2024 May 8;48(4):2775–82. doi: 10.1007/s11259-024-10405-0 (PMC11315736; doi:10.1007/s11259-024-10405-0)
Supplement: Supplementary file 1 — Supplementary Material 1 [file 11259_2024_10405_MOESM1_ESM.docx]

**
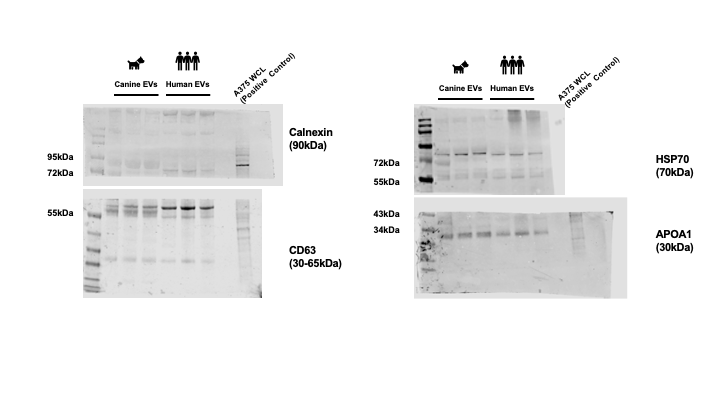
**

**Supplementary Figure 1 – Uncropped Western Blot Images**

Uncropped Western Blot Images as seen in Figure 4. Membranes cut prior to incubation with primary antibody, and WCL removed from HSP70 membrane due to signal interference prior to developing.


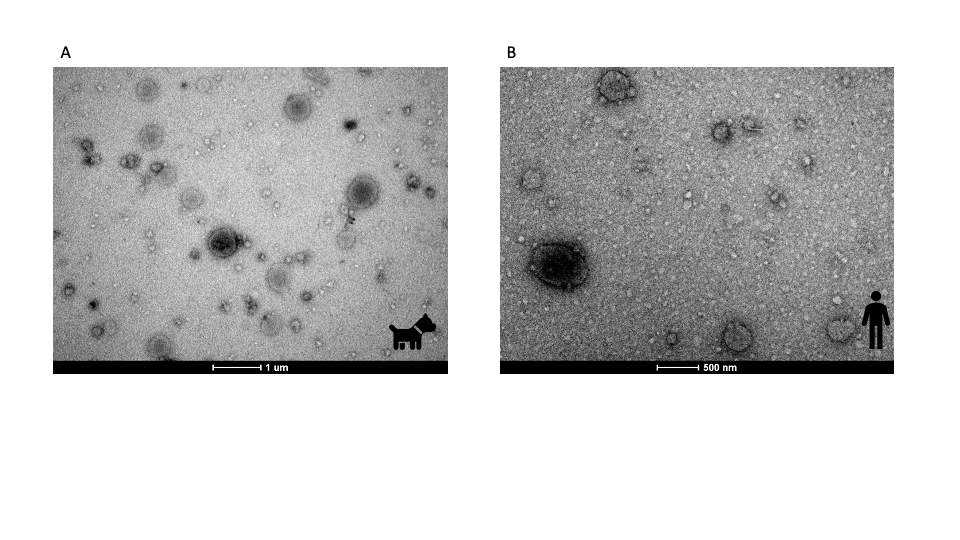


**Supplementary Figure 2: Wide-field Transmission Electron Microscopy Images of Canine and Human Plasma-derived EVs**

TEM images taken of **(A)** canine EVs at 11500x and **(B)** human EVs at 20500x show a wide-field view of plasma-derived EVs, with associated lipoproteins and larger microvesicles.

**Supplementary Table 1 – Demographic Details of Human Plasma Donors**

| **Donor Age** | **Biological Sex** | **Known Medical Conditions** |
| --- | --- | --- |
| 34 | Female | None |
| 34 | Female | None |
| 32 | Female | None |
